# Supplementary material for: Salmonella enters a dormant state within human epithelial cells for persistent infection
Source: PLoS Pathog. 2021 Apr 30;17(4):e1009550. doi: 10.1371/journal.ppat.1009550 (PMC8115778; doi:10.1371/journal.ppat.1009550)
Supplement: S1 Table — (DOCX) [file ppat.1009550.s001.docx]

**S1 Table. *S.* Typhimurium strains used in this study**

| **Name** | **Genotype** | **Plasmid** | **Reference** |
| --- | --- | --- | --- |
| **JL129** | **SL1344 Wild type** | **pSINA1.1** | **This study** |
| **JL147** | **SL1344 Wild type** | **pSINA1.4** | **This study** |
| **JL148** | **SL1344 Wild type** | **pSINA1.5** | **This study** |
| **JL177** | **SL1344 Wild type** | **pSINA1.7** | **This study** |
| **JL179** | **SL1344 Wild type** | **pSINA1.9** | **This study** |
| **JL171** | **SL1344 *ΔssrB*** | **pSINA1.1** | **This study** |
| **JL173** | **SL1344 *ΔssaV*** | **pSINA1.1** | **This study** |
| **JL158** | **SL1344 *Δlon*** | **pSINA1.1** | **This study** |
| **JL180** | **SL1344 *ΔdksA*** | **pSINA1.9** | **This study** |
| **JL185** | **SL1344 *ΔrelA*** | **pSINA1.9** | **This study** |
| **JL181** | **SL1344 *ΔrelA ΔspoT*** | **pSINA1.9** | **This study** |
| **JL182** | **SL1344 Wildtype *pipB2::HA*** | **pSINA1.7** | **This study** |
