# Supplementary material for: Salmonella enters a dormant state within human epithelial cells for persistent infection
Source: PLoS Pathog. 2021 Apr 30;17(4):e1009550. doi: 10.1371/journal.ppat.1009550 (PMC8115778; doi:10.1371/journal.ppat.1009550)
Supplement: S2 Table — (DOCX) [file ppat.1009550.s002.docx]

**S2 Table. Plasmids used in this study**

| **Name** | **Description** | **Reference** |
| --- | --- | --- |
| **pBR322 Timer^bac^** | **Timer^bac^ expression** | **[1]** |
| **pM973** | **SPI-2 activity of *S.* Typhimurium** | **[2]** |
| **pTSAR1** | ***Shigella* T3SS activity** | **[3]** |
| **pHRdSV40-NLS-dCas9-24xGCN4_v4-NLS-P2A-BFP-dWPRE** | **Template of tagBFP** | **[4]** |
| **pBAD smURFP-HO-1** | **Inducible expression of smURFP** | **[5]** |
| **pPssaG-tagBFP** | **SPI-2 activity of *S.* Typhimurium** | **This study** |
| **pPuhpT-smURFP** | **Cytosolic access of *S.* Typhimurium** | **This study** |
| **pSINA-int** | **Subcloning PssaG-tagBFP and PuhpT-smURFP** | **This study** |
| **pSINA1.1** | **Detection of *S.* Typhimurium lifestyles** | **This study** |
| **pSINA1.4** | **Detection of *S.* Typhimurium lifestyles using immunofluorescence** | **This study** |
| **pSINA1.5** | **Detection of *S.* Typhimurium lifestyles with inducible smURFP expression** | **This study** |
| **pSINA1.7** | **Detection of *S.* Typhimurium lifestyles** | **This study** |
| **pBAD hilA** | **Inducible expression of *hil*A** | **This study** |
| **pSINA1.9** | **Detection of *S.* Typhimurium lifestyles with inducible *hil*A expression** | **This study** |

**References**

1. Claudi B, Spröte P, Chirkova A, Personnic N, Zankl J, Schürmann N, et al. Phenotypic variation of salmonella in host tissues delays eradication by antimicrobial chemotherapy. Cell. 2014;158: 722–733. doi:10.1016/j.cell.2014.06.045
2. Hapfelmeier S, Stecher B, Barthel M, Kremer M, Müller AJ, Heikenwalder M, et al. The Salmonella Pathogenicity Island (SPI)-2 and SPI-1 Type III Secretion Systems Allow Salmonella Serovar typhimurium to Trigger Colitis via MyD88-Dependent and MyD88-Independent Mechanisms . J Immunol. 2005;174: 1675–1685. doi:10.4049/jimmunol.174.3.1675
3. Campbell-Valois FX, Schnupf P, Nigro G, Sachse M, Sansonetti PJ, Parsot C. A fluorescent reporter reveals on/off regulation of the shigella type III secretion apparatus during entry and cell-to-cell spread. Cell Host Microbe. 2014;15: 177–189. doi:10.1016/j.chom.2014.01.005
4. Tanenbaum ME, Gilbert LA, Qi LS, Weissman JS, Vale RD. A protein-tagging system for signal amplification in gene expression and fluorescence imaging. Cell. 2014;159: 635–646. doi:10.1016/j.cell.2014.09.039
5. Rodriguez EA, Tran GN, Gross LA, Crisp JL, Shu X, Lin JY, et al. A far-red fluorescent protein evolved from a cyanobacterial phycobiliprotein. Nat Methods. 2016;13: 763–769. doi:10.1038/nmeth.3935
