# Supplementary material for: Salmonella enters a dormant state within human epithelial cells for persistent infection
Source: PLoS Pathog. 2021 Apr 30;17(4):e1009550. doi: 10.1371/journal.ppat.1009550 (PMC8115778; doi:10.1371/journal.ppat.1009550)
Supplement: S3 Table — (DOCX) [file ppat.1009550.s003.docx]

**S3 Table. Primers used for molecular cloning in this study**

| **Name** | **Sequence** | **Purpose** |
| --- | --- | --- |
| **tagBFP_fw** | **GAATTCAGGAGGTAGTATTGatgagcgagctgattaaggag** | **Amplification of *tagBFP*** |
| **tagBFP_rv** | **ATCttaattaagcttgtgccccag** | **Amplification of *tagBFP*** |
| **uhpT_fw** | **GAATTCcgcgagacccacgaagcgtg** | **Amplification of *uhpT* promoter** |
| **uhpT_rv** | **GGATCCCATggattactcctgagc** | **Amplification of *uhpT* promoter** |
| **smURFP_fw** | **ggatccAAAACTTCTGAACAACGTGTAAACATCGC** | **Amplification of *smURFP*** |
| **smURFP_rv** | **tctagaCTAGCCTTCGGAGGTGGCgag** | **Amplification of *smURFP*** |
| **Vac_fw** | **TCTAGACGGTAGATTAGCCTTAACCGCg** | **Amplification of vacuolar module** |
| **Cyt_rv** | **gcatgcGTAAAACGACGGCCAGTGCC** | **Amplification of cytosolic module** |
| **GFP_fw** | **tctagatttaagaaggagatatacatATGAGTAAAGGAGAAGAACTTTTCACTGGA** | **Amplification of *GFP*** |
| **GFP_rv** | **AAGCTTTTATTTGTATAGTTCATCCATGCC** | **Amplification of *GFP*** |
| **Ara_fw** | **GTCGACatgtgcctgtcaaatggacg** | **Amplification of inducible *smURFP* cassette** |
| **Ara_rv** | **GCATGCGTAGAAACGCAAAAAGGCCATCCG** | **Amplification of inducible *smURFP* cassette** |
| **DsRed_fw** | **gtccacgtagtagtagccgggc** | **Mutagenesis of *Timer^bac^* to *DsRed*** |
| **DsRed_rv** | **tccaagctggacatcacctccc** | **Mutagenesis of *Timer^bac^* to *DsRed*** |
| **hilA_fw1** | **GGATCCatgccacattttaatcctgttcc** | **Amplification of *hilA*** |
| **hilA_rv1** | **gtttAAACttaccgtaatttaatcaagcggggGtcctgtttccatcttttgaacc** | **Amplification of *hilA*** |
| **hilA_fw2** | **atctaaGTCGACatgtgcctgtcaaatggacg** | **Amplification of inducible *hilA* cassette** |
| **hilA_rv2** | **gtagaaacgcaaaaaggccatccg** | **Amplification of inducible *hilA* cassette** |
