# Supplementary material for: Salmonella enters a dormant state within human epithelial cells for persistent infection
Source: PLoS Pathog. 2021 Apr 30;17(4):e1009550. doi: 10.1371/journal.ppat.1009550 (PMC8115778; doi:10.1371/journal.ppat.1009550)
Supplement: S4 Table — (DOCX) [file ppat.1009550.s004.docx]

**S4 Table. Antibodies used in this study**

| **Name of reagent** | **Concentration** | **Supplier** | **Reference** |
| --- | --- | --- | --- |
| **Anti-RAB5A antibody** | **1:200** | **ProteinTech** | **20228-1-AP** |
| **Anti-RAB7 antibody** | **1:200** | **Sigma** | **R4779** |
| **Anti-RAB11A antibody** | **1:200** | **Invitrogen** | **3H18L5** |
| **Anti-LAMP1 antibody** | **1:200** | **abcam** | **ab19294** |
| **Anti-LC3B antibody** | **1:200** | **abcam** | **ab51520** |
| **Anti-*S.* Typhimurium antibody** | **1:200** | **abcam** | **ab35156** |
| **Anti-HA antibody** | **1:200** | **Sigma** | **H6908** |
| **Goat anti-Rabbit IgG (H+L) Highly Cross-Adsorbed Secondary Antibody, Alexa Fluor 488** | **1:200** | **Invitrogen** | **A11034** |
| **Goat anti-Rabbit IgG (H+L) Cross-Adsorbed Secondary Antibody, Cyanine5** | **1:200** | **Invitrogen** | **A10523** |
| **Rhodamine Phalloidin** | **1:100** | **Invitrogen** | **R415** |
