# Supplementary figures and images for: Salmonella enters a dormant state within human epithelial cells for persistent infection
Source: PLoS Pathog. 2021 Apr 30;17(4):e1009550. doi: 10.1371/journal.ppat.1009550 (PMC8115778; doi:10.1371/journal.ppat.1009550)

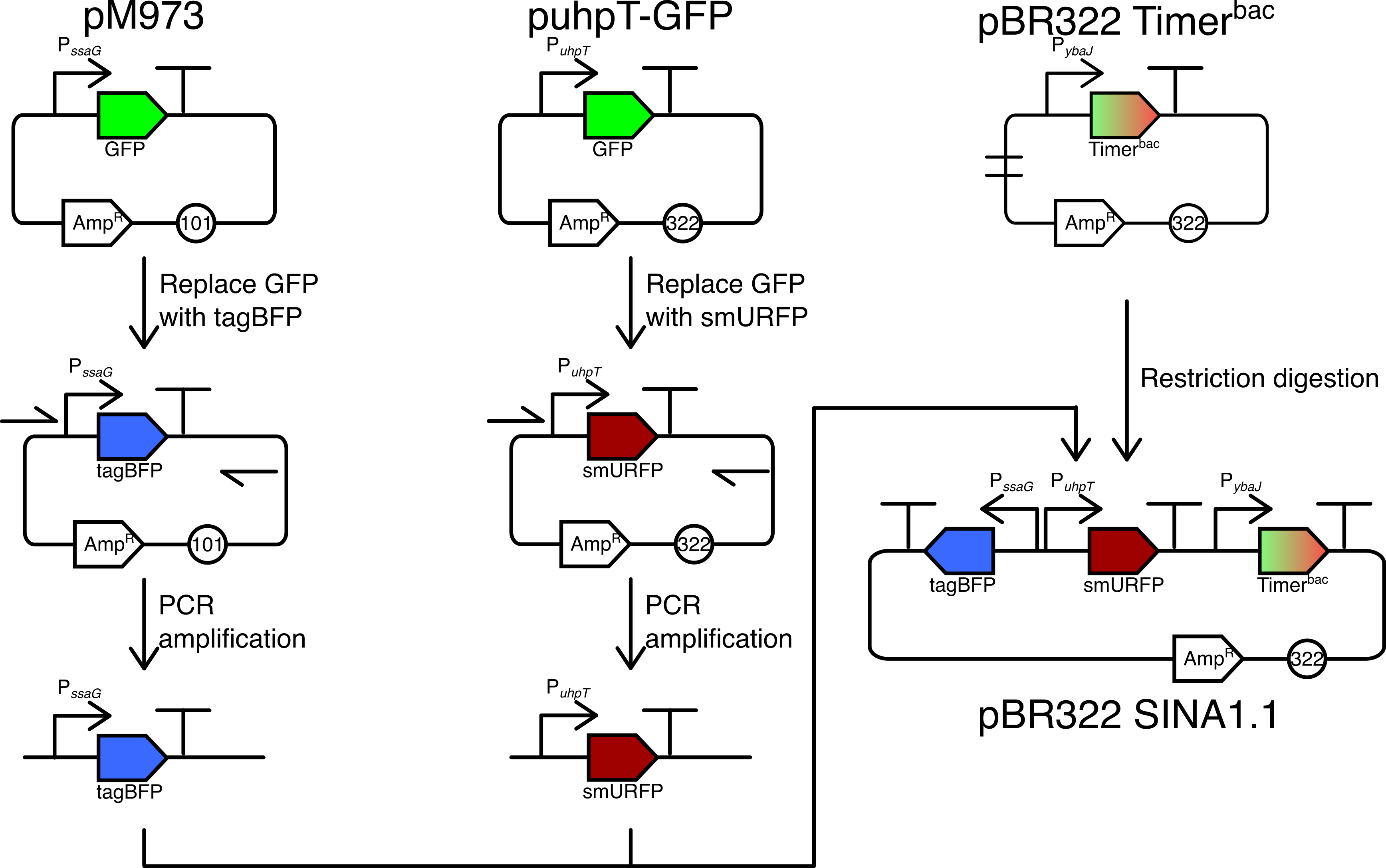

Supplement: S1 Fig — The vacuolar and cytosolic modules were first individually tested with GFP (pM973 and puhpT-GFP), and then switched to tagBFP and smURFP, respectively. The vacuolar (PssaG-tagBFP) and cytosolic (PuhpT-smURFP) modules were subsequently amplified and introduced into pBR322 Timerbac between SphI and SalI sites to yield SINA1.1. (TIF) [file ppat.1009550.s005.tif]

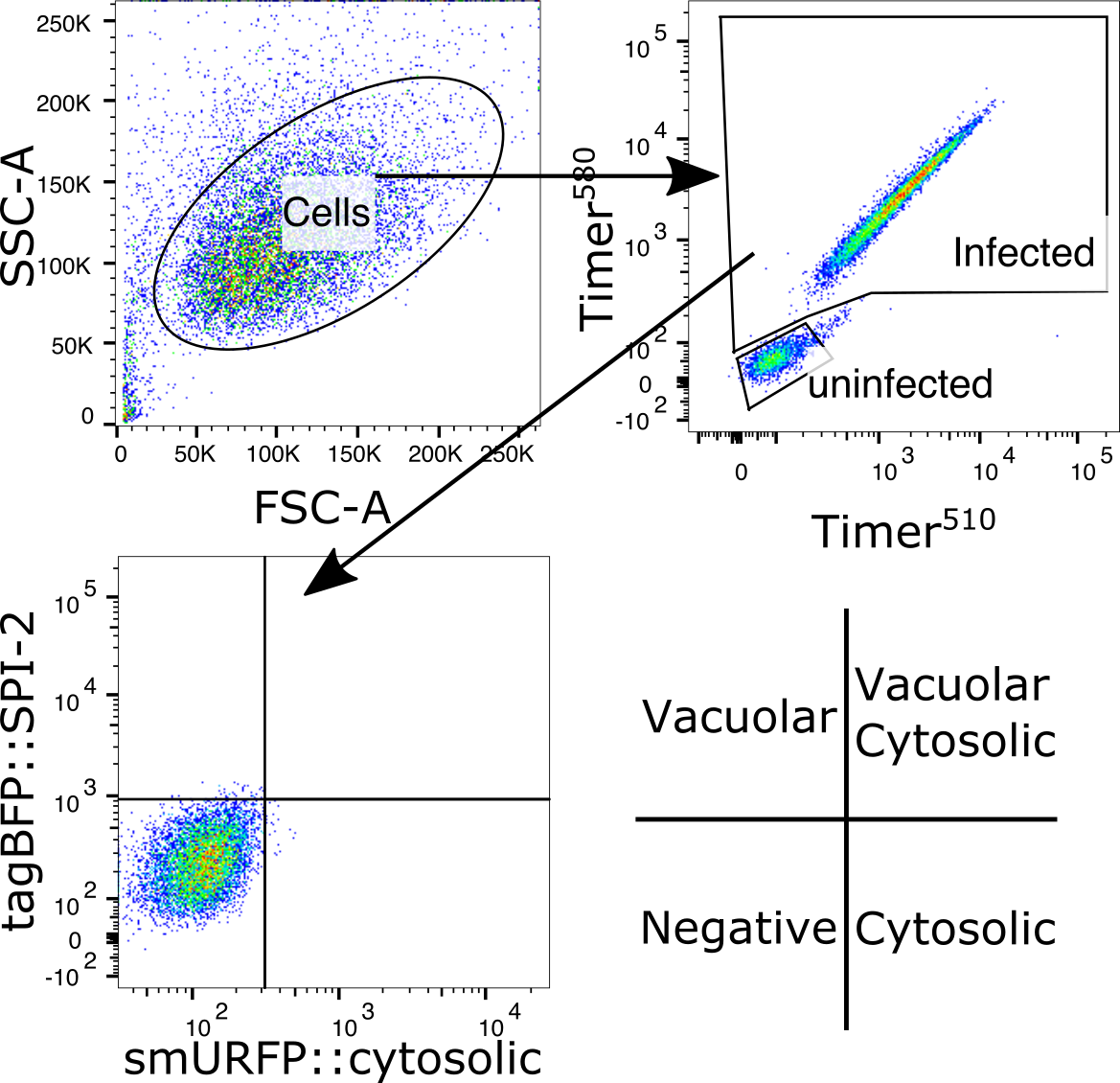

Supplement: S2 Fig — Analyzed events were first gated for “Cells” on SSC-A vs FSC-A plot to remove cell debris. In the “Cells” events, “Uninfected” population was gated by double-negative; “Infected” was gated by double-positive on Timer580 vs Timer510 plot. To gate for the basal intensity of SINA1.1 at 1 h pi, four quadrants were drawn in the “Infected” events on tagBFP::SPI-2 vs smURFP::cytosolic plot, where the biological interpretations of the four quadrants were denoted in the bottom-right sketch. (TIF) [file ppat.1009550.s006.tif]

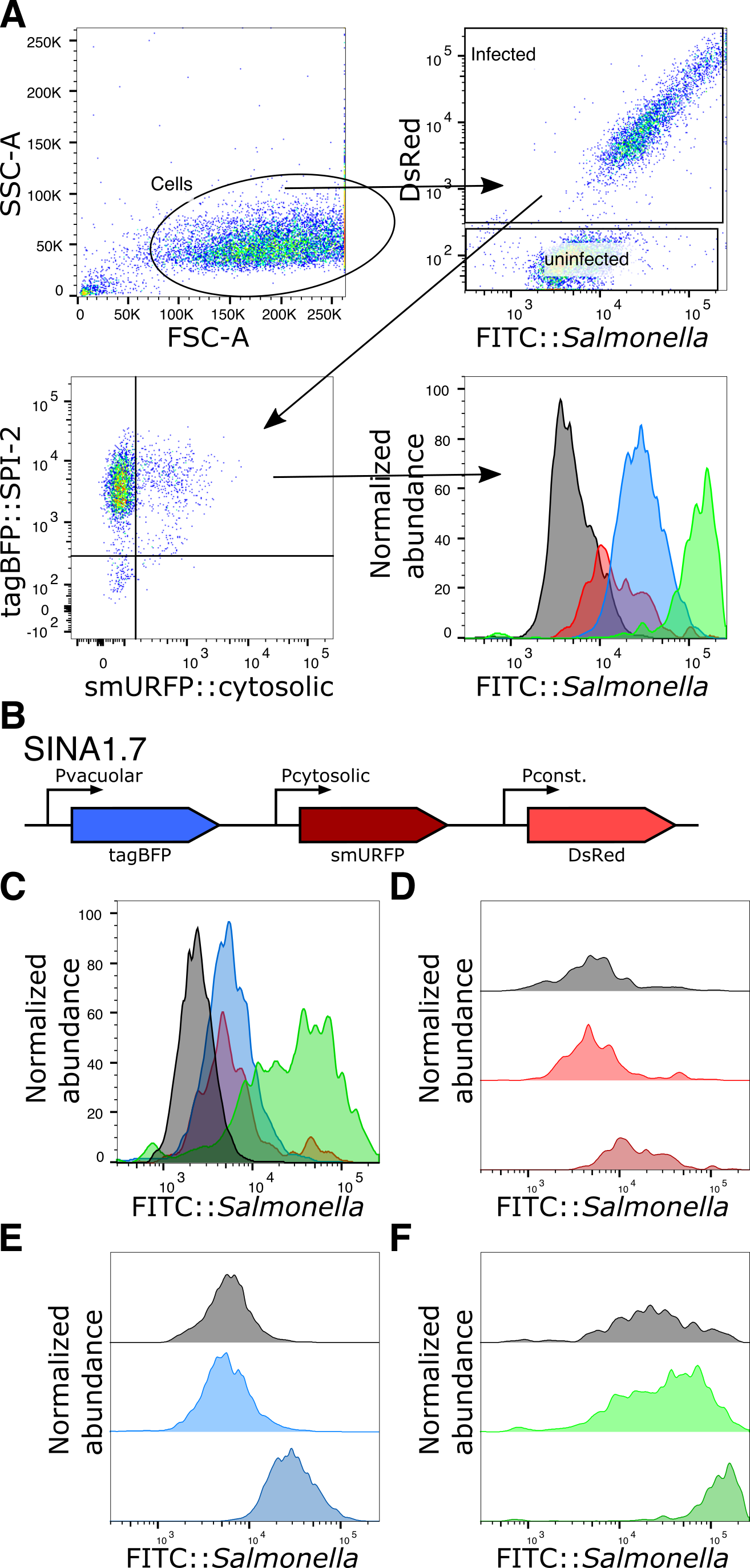

Supplement: S3 Fig — (A) Gating strategy for applying SINA1.7 for digitonin assay. HeLa cells were infected with SINA1.7-harboring wild type S. Typhimurium, and harvested at 6 h pi for analysis by flow cytometry. The events were first gated for “Cells” to remove cell debris and subsequently gated for “uninfected” and “infected” based on DsRed signal. The “infected” events were subsequently gated for Vac-Cyt-, Vac+Cyt- and Vac+Cyt+ on tagBFP::SPI-2 vs smURFP::cytosolic plot. The fluorescence profiles FITC::S. Typhimurium (after immunostaining using anti-S. Typhimurium antibody) of Vac-Cyt-, Vac+Cyt- and Vac+Cyt+ and “uninfected” were plotted as overlay histograms. The gating strategy displays a positive control sample treated with saponin. (B) Schematic diagram for the constructions of the SINA derivative SINA1.7, where Timerbac was replaced with DsRed as compared to SINA1.1. (C) Digitonin assay on SINA-1.7 harboring wild type S. Typhimurium-infected HeLa cells at 6 h pi, signal intensities of uninfected (black), Vac-Cyt- (red), Vac+Cyt- (blue) and Vac+Cyt+ (green) populations immunostained against anti-S. Typhimurium. (D) Digitonin assay on SINA-1.7 harboring wild type S. Typhimurium infected HeLa cells at 6 h pi, signal intensity of Vac-Cyt- population unpermeabilized (black, negative control), permeabilized with digitonin (red) and saponin (maroon, positive control). (E) Digitonin assay on SINA-1.7 harboring wild type S. Typhimurium infected HeLa cells at 6 h pi, signal intensity of Vac+Cyt- population unpermeabilized (black, negative control), permeabilized with digitonin (blue) and saponin (navy, positive control). (F) Digitonin assay on SINA-1.7 harboring wild type S. Typhimurium infected HeLa cells at 6 h pi, signal intensity of Vac+Cyt+ population unpermeabilized (black, negative control), permeabilized with digitonin (green) and saponin (dark Green, positive control). (TIF) [file ppat.1009550.s007.tif]

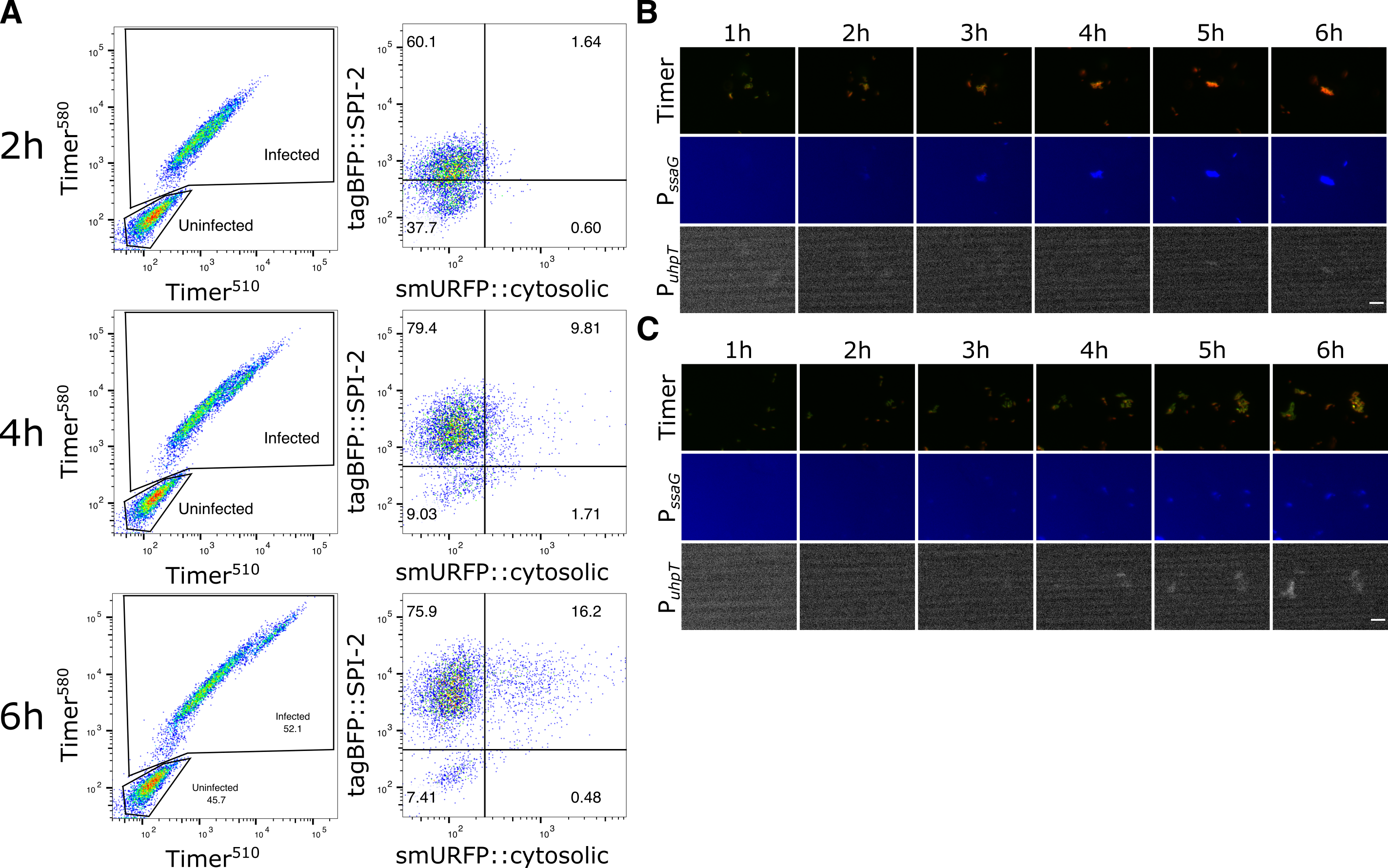

Supplement: S4 Fig — HeLa cells were infected with wild type S. Typhimurium harboring SINA1.1. (A) Infected cells were harvested and analyzed at time intervals of 2 h, 4 h and 6 h pi. (Left) Timerbac profile of total cells at 2 h (top), 4 h (middle) and 6 h (bottom) pi in HeLa cells. (Right) Fluorescence output of the localization module of infected cells at 2 h (top), 4 h (middle) and 6 h (bottom) pi in HeLa cells. (B-C) Time-lapse microscopic acquisition of the S. Typhimurium intracellular lifestyle. Representative images of SINA1.1 signal output of vacuolar (B) and cytosolic (C) S. Typhimurium. Scare bars are 10 μm. (TIF) [file ppat.1009550.s008.tif]

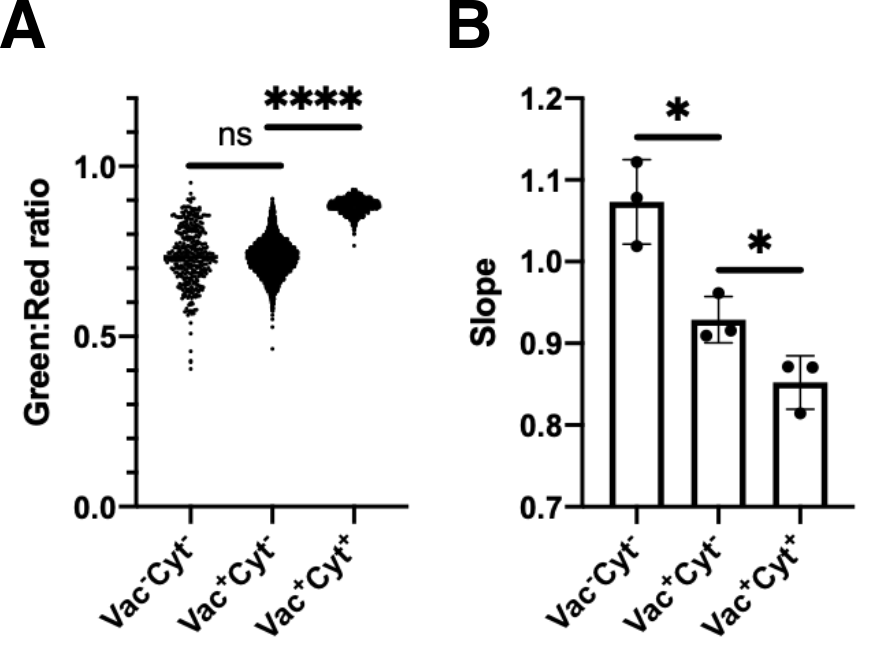

Supplement: S5 Fig — HeLa cells were infected with SINA1.1-harboring S. Typhimurium and harvested at 6 h pi for analysis by flow cytometry. The three infected cell populations, Vac-Cyt-, Vac+Cyt- and Vac+Cyt+ on tagBFP::SPI-2 vs smURFP::cytosolic plot were backgated on Timer580 vs Timer510 plot. Timer580 and Timer510 intensities were extracted from each event. (A) Quantification of Green:red ratio of Vac-Cyt-, Vac+Cyt- and Vac+Cyt+ population in Timerbac plot at 6 h pi. Green:red ratios were calculated by dividing Timer510 by Timer580 values, and plotted against infected cell populations. (B) Quantification of the slope of the best-fitted line of Vac-Cyt-, Vac+Cyt- and Vac+Cyt+ population in Timerbac plot at 6 h pi. For each population, a best-fitted line was plotted on the Timer580 vs Timer510 plot to extract the slopes for each infected cell populations. At least a total of 1000 events of infected cells were analyzed by flow cytometry in triplicate experiments. The bars represent the mean value, unpaired t-tests were carried out, *P < 0.05, ****P < 0.0001, ns: not significant. (TIF) [file ppat.1009550.s009.tif]

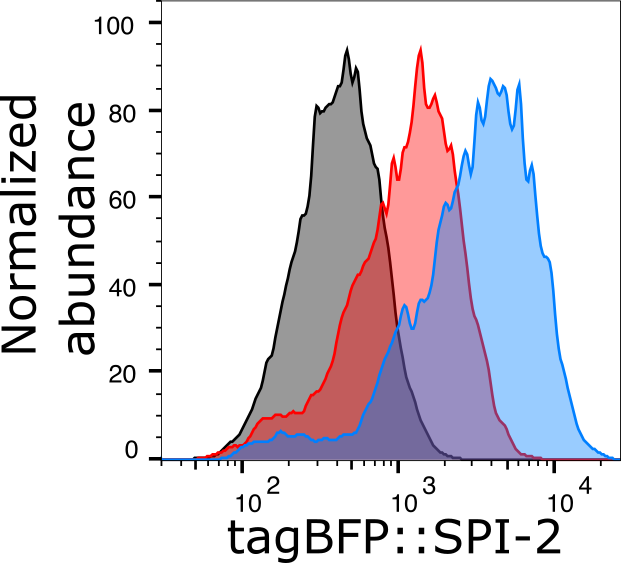

Supplement: S6 Fig — HeLa cells were infected with SINA1.1-harboring S. Typhimurium, and harvested at 1 h, 2 h and 3 h pi for analysis by flow cytometry. The infected cells were gated and the fluorescence profiles of vacuolar submodule PssaG-tagBFP at 1 h (black), 2 h (red) and 3 h (blue) pi were plotted as overlaying histograms. (TIF) [file ppat.1009550.s010.tif]

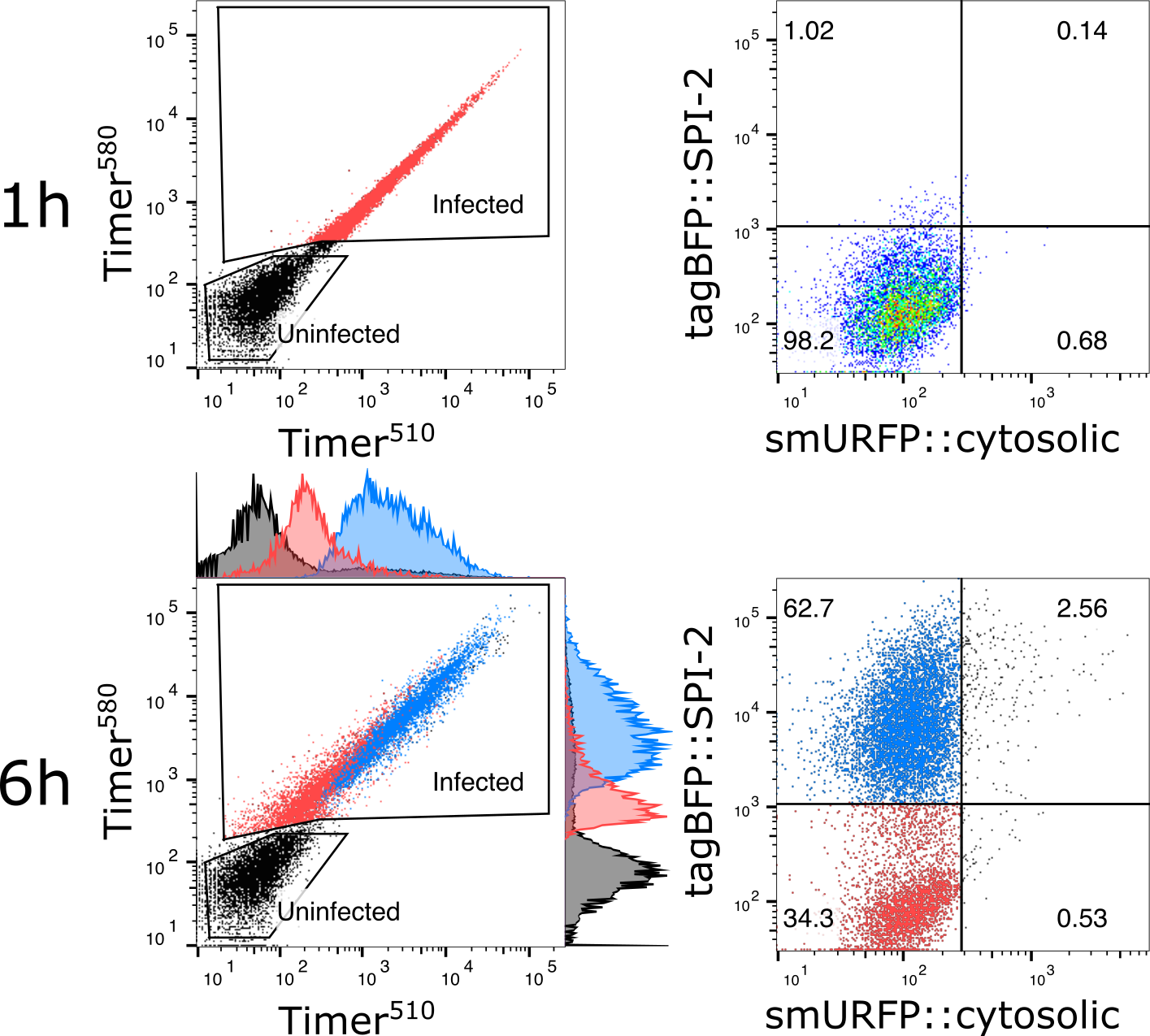

Supplement: S7 Fig — Polarized Caco-2 monolayers were infected with SINA1.1-harboring S. Typhimurium and harvested at 1 h and 6 h pi for analysis by flow cytometry. (Left) Timerbac profile of Vac-Cyt- (red) and Vac+Cyt- (blue) populations and total cells (black) at 1 h (top) and 6 h (bottom) pi in Caco-2 cells. (Right) Distribution of Vac-Cyt- and Vac+Cyt- populations at 1 h (top) and 6 h (bottom) pi in polarized Caco-2 cells. (TIF) [file ppat.1009550.s011.tif]

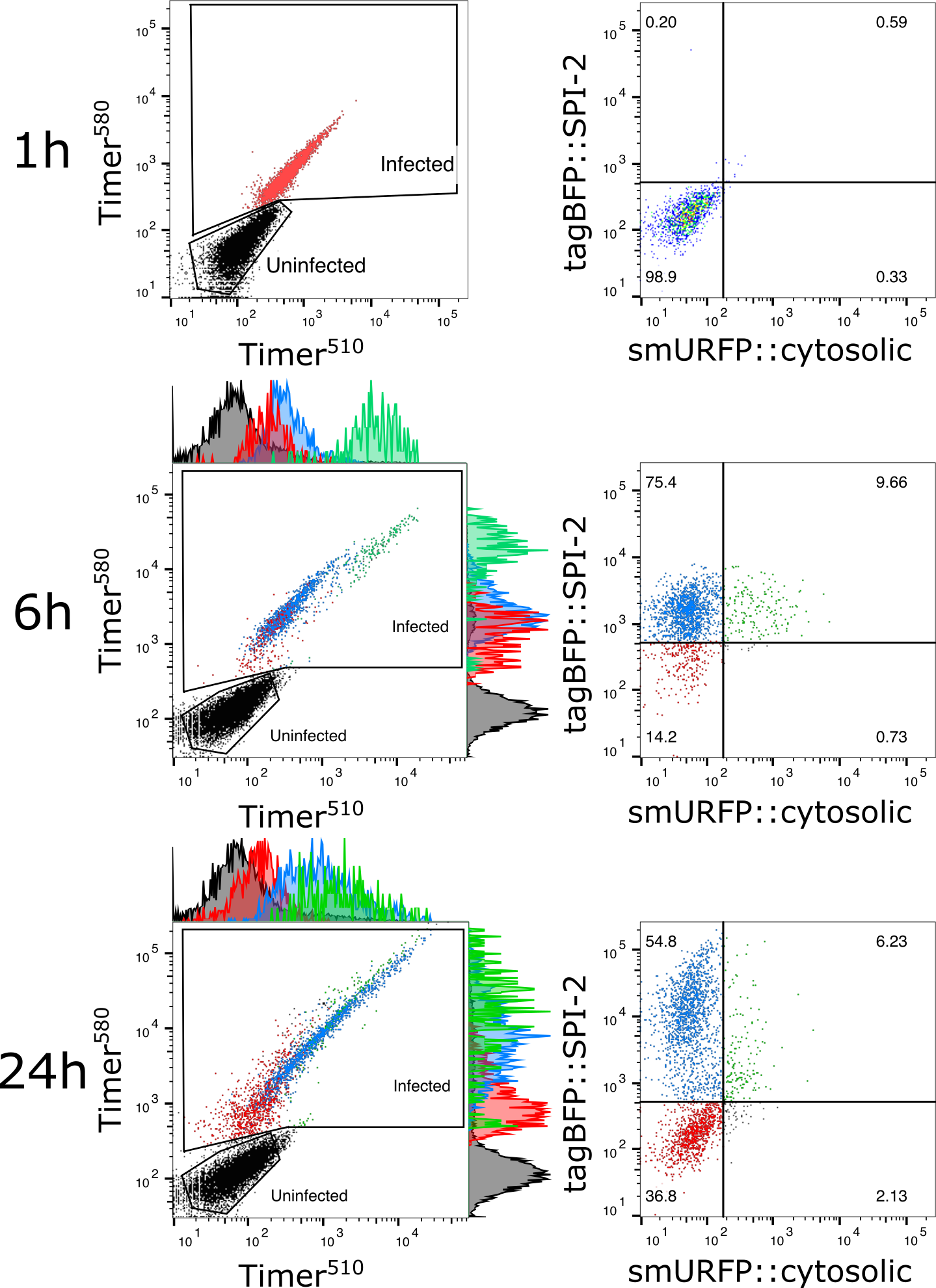

Supplement: S8 Fig — 3T3 cells were infected with SINA1.1-harboring S. Typhimurium and harvested at 1 h, 6 h and 24 h pi for analysis by flow cytometry. (Left) Timerbac profile of Vac-Cyt- (red), Vac+Cyt- (blue) and Vac+Cyt+ (green) populations and total cells (black) at 1 h (top), 6 h (middle) and 24 h (bottom) pi in 3T3 cells. (Right) Distribution of Vac-Cyt-, Vac+Cyt+ and Vac+Cyt+ populations at 1 h (top), 6h (middle) and 24 h (bottom) pi in 3T3 cells. (TIF) [file ppat.1009550.s012.tif]

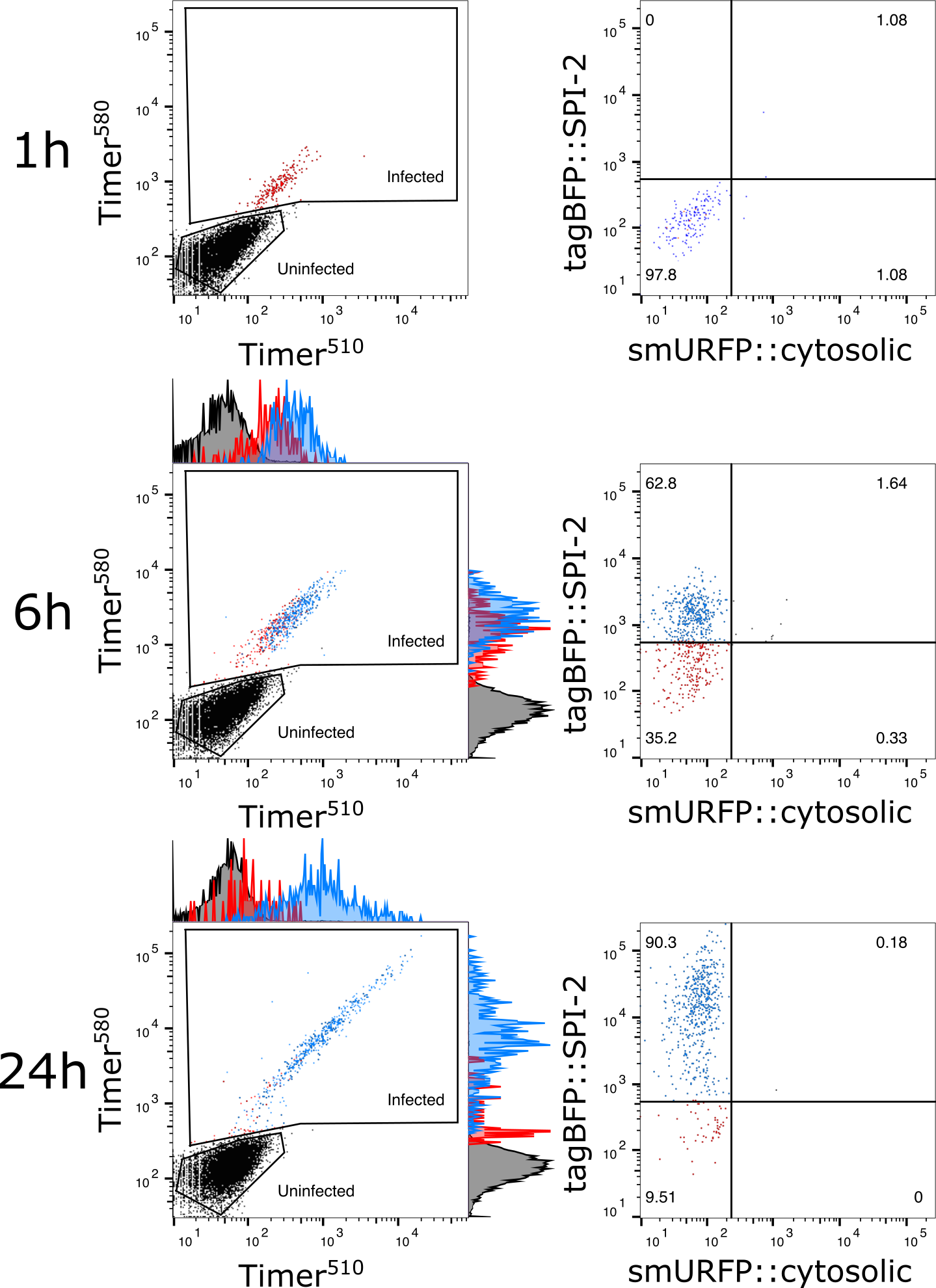

Supplement: S9 Fig — Differentiated THP-1 cells were infected with SINA1.1-harboring S. Typhimurium and harvested at 1 h, 6 h and 24 h pi for analysis by flow cytometry. (Left) Timerbac profile of Vac-Cyt- (red) and Vac+Cyt- (blue) populations and total cells (black) at 1 h (top), 6 h (middle) and 24 h (bottom) pi in THP-1 cells. (Right) Distribution of Vac-Cyt- and Vac+Cyt- populations at 1 h (top), 6 h (middle) and 24 h (bottom) pi in differentiated THP-1 cells. (TIF) [file ppat.1009550.s013.tif]

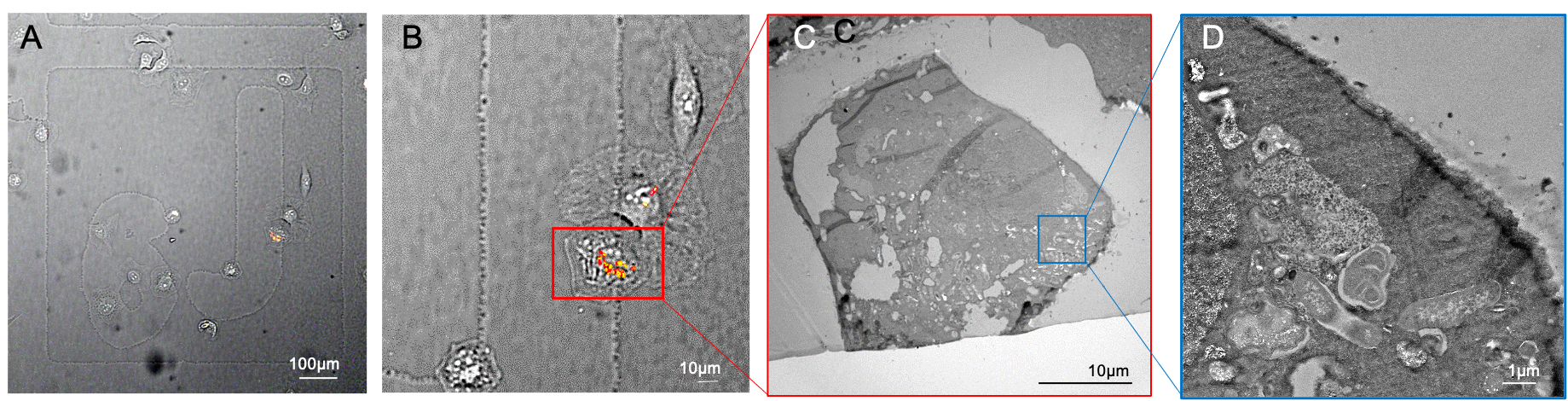

Supplement: S10 Fig — (A) Brightfield and fluorescent microscopy image of region of interest on MatTek dish. (B) Brightfield and fluorescent microscopy image of cells of interest harboring Vac-Cyt- S. Typhimurium. (C) TEM image of cell of interest in labelled region from (B). (D) Magnified TEM image of labelled region from (C). (TIF) [file ppat.1009550.s014.tif]

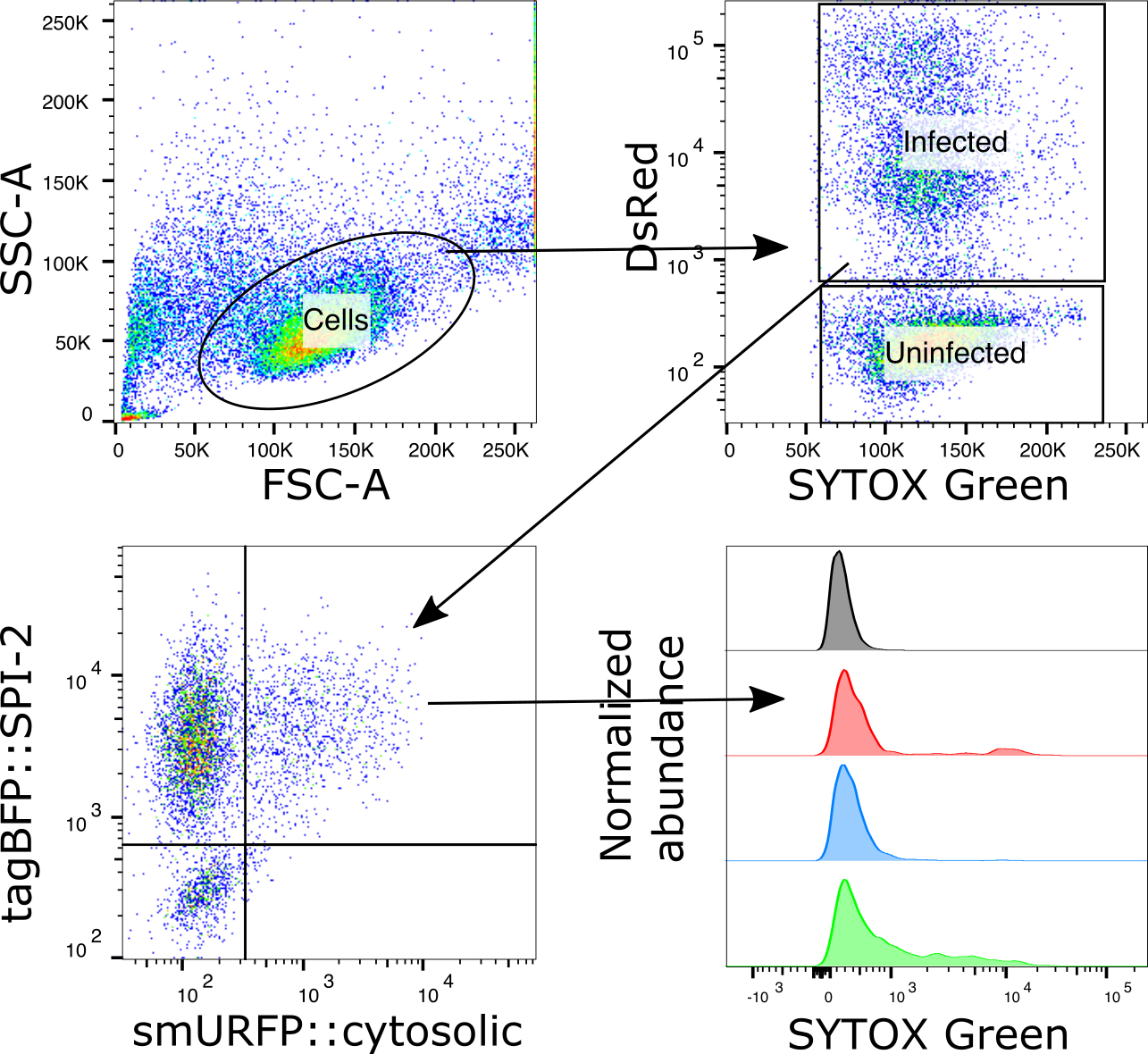

Supplement: S11 Fig — HeLa cells were infected with SINA1.7 harboring S. Typhimurium, harvested at 6 h pi and stained with SYTOX Green and analyzed by flow cytometry. The infected cells were gated and the fluorescence profiles of SYTOX Green in uninfected cell (black), Vac-Cyt- (red), Vac+Cyt- (blue) and Vac+Cyt+ (green) were plotted as offset histograms. (TIF) [file ppat.1009550.s015.tif]

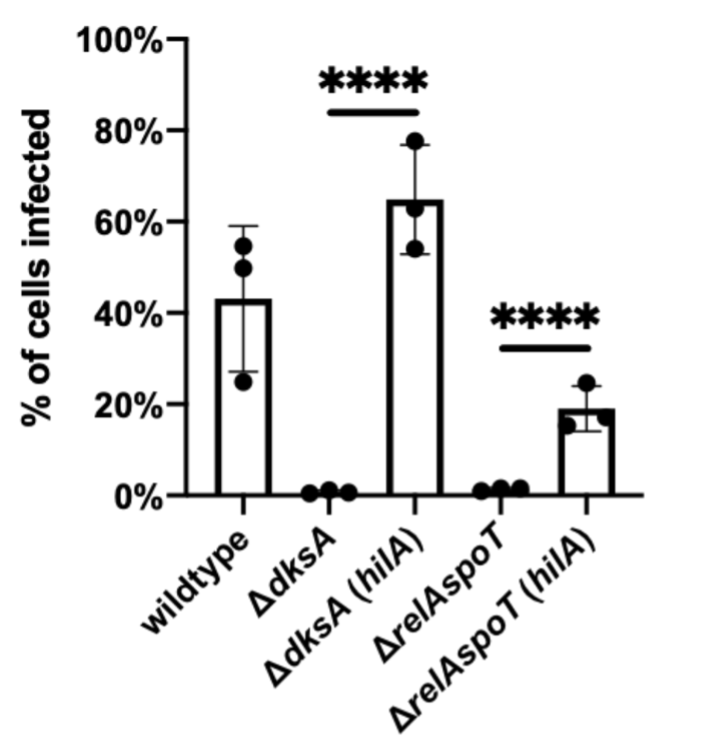

Supplement: S12 Fig — HeLa cells were infected with various S. Typhimurium strains and harvested at 6 h pi for flow cytometry analysis. The losses of invasiveness in ΔdksA and ΔrelAspoT mutants are rescued by ectopic expression of hilA from the arabinose inducible cassette in SINA1.9. At least a total of 1000 events of infected cells were analyzed by flow cytometry in triplicate experiments. The bars represent the mean value, unpaired t-test was carried out, ****P < 0.0001. (TIF) [file ppat.1009550.s016.tif]
