# Supplementary material for: Salmonella enters a dormant state within human epithelial cells for persistent infection
Source: PLoS Pathog. 2021 Apr 30;17(4):e1009550. doi: 10.1371/journal.ppat.1009550 (PMC8115778; doi:10.1371/journal.ppat.1009550)
Supplement: Attachment — Submitted filename: LukEtAl_ResponseReviewers_Final_2403_cleaned.docx [file ppat.1009550.s020.docx]

Responses to Reviewers’ Questions

We would like to express our gratitude to the Reviewers for their time to provide feedback that helped to improve the manuscript. We have carefully taken into consideration all the suggestions and the feedback from the reviewers. Our responses to tthe individual comments are displayed in blue in this letter.

We would like to mention that it has been logistically challenging to perform this revision in light of the COVID crisis requiring us to bring in the first author safely from the UK to France. It required a huge amount of administrative work, and also caused quarantine delays; so we do hope that all these efforts are recognized by the reviewers. Also, we needed to mobilize additional staff members leading to a longer list of co-authors.

**Part I - Summary**

Reviewer #1: In this manuscript by Luk et al., the authors construct a new tool (SINA) to characterize distinct populations of Salmonella within enterocytes. They use SINA to identify subpopulations of intracellular Salmonella and describe a population of bacteria that are not within the “classical” SCV or within the cytosol, but seem to reside in an uncharacterized vesicular compartment in a dormant state. The dormant bacteria do not express SPI2 at 6 hours post-infection, but appear to express it at later time points. Finally, they propose that the dormant state is regulated by (p)ppGpp-stringent response through SpoT. The authors propose that dormant Salmonella within epithelial cells mediate persistence. This is a potentially interesting study, however, additional data and controls are needed. The manuscript would benefit from clearly defining at the beginning what dormancy is and how does this compare to persisters.

We would like to thank Reviewer #1 for the time in evaluating our manuscript and we appreciate the comments on the novelty of our experimental tools and the importance of our work. We have managed to perform substantial additional characterization and controls (detailed below), and we hope that these new controls and additional data, plus the changes to the text will be considered an improvement to the previous version of the manuscript. Following the comment on a definition of dormancy, we have included an explicit description of dormancy and comparison with persistence in the introduction section (Lines 41-44).

**Part II – Major Issues: Key Experiments Required for Acceptance**

Reviewer #1: 1) the authors claim that the dormant state mediates persistence, but they don’t really show this. First off, they need to define what they mean by persistence. The data presented in Figure 4 are not very convincing. They only show 6 h, 24 h, and 168 h. What happens at times in between? They see that ~50% of the “dormant” bacteria begin to express SPI2 at about 24 hours. Do these bacteria replicate, burst out and reinfect neighboring epithelial cells to start the cycle again? Showing images of these longer infections and more time points in between 1 and 7 days would help sort this out.

We thank the comments given by Reviewer #1. We have clarified the definition of persistence in the manuscript (line 41-44). We have added data profiling the bacterial behavior over the 7 day time course using flow cytometry referring to figure 2F. This was done as it enables the extraction of much more information as compared to microscopy. We enriched the infected HeLa cells harboring dormant *S*. Typhimurium as described in Materials and Methods, the collected cells were cultured in DMEM supplemented with Gen^10^. This treatment is important as extracellular bacteria released from the infected cells are killed efficiently through this approach, and reinfection is inhibited. At every timepoint, we harvested the cells and analyzed by flow cytometry, following the gating strategy described in Fig S2. Here, we plot the SPI-2 expression profile of infected cells from day 1 to day 7 after the enrichment of dormant *S*. Typhimurium-harboring cells at 6 h pi, where we observed a gradual reduction of SPI-2-expressing population. As the figure is already very complex, we would prefer to show this data only to the reviewers instead of making the figure even more heavy.

Figure: Histograms plotting the SPI-2 expression profile of infected cells from day 1 to day 7 after the enrichment of dormant *S*. Typhimurium-harboring cells at 6 h pi.


2) Movies and images need to show host cells with either phase of DIC imaging. For example, in movie 3, I see some red and green fluorescence, but I have no idea whether they are inside of host cells or extracellular.

We thank the suggestion offered by Reviewer #1. We have included the brightfield channel for the time lapse microscopy movies. We would like to point out that that the fluorescence focus is different from the one in the brightfield channel, and we have not had the automated z offset module available during this revision. Therefore, we cannot obatain an optimal brightfield focus, as we would not see the individual bacteria.

3) I’d like to see better characterization of the “unique” vesicular compartment that they claim dormant Salmonella reside in. Fig.3 only shows 1 time point, 6 h and I don’t know what “minor” recruitment of LAMP1 means. Can this be quantitated over a time course? Also, transmission electron microscopy would be nice confirmation that these bacteria are really in a vesicle of some sort.

We agree with the suggestion by Reviewer #1, and we have included additional characterization on the dormant SCV by immunostaining the compartment with early and intermediate SCV markers, including RAB5, RAB7 and RAB11 (Figure 2D and 2E). It appears that despite all our efforts this compartment still requires further characterization, however this is beyond the scope of this study, and will be tackled in an ongoing piece from our team that uses comprehensive proteomics on the compartment containing dormant *Salmonella*. We have noted that in the manuscript and also refer to the knowledge of the vesicular *Shigella* compartment that is still poorly characterized despite more than two decades of research.

Furthermore, we have also performed a detailed correlative light electron microscopy (CLEM) study using serial sections (to monitor the membranes around the dormant Salmonella) to double confirm the vesicular localization of dormant *S*. Typhimurium (Figure 2B). In addition, we also performed CLEM via FIB-SEM, however our FIB-SEM system encountered technical difficulties during the course of the revision, and it was difficult to get the machine fixed in the current situation. Therefore, we decided to use the TEM data from sections. We found that RAB5, RAB7 and RAB11 did not localize at the dormant SCV (Figure 2E).

**Part III – Minor Issues: Editorial and Data Presentation Modifications**

Reviewer #1: 1) many sentences don’t make sense. I will give one example of many, e.g. on page 14, first paragraph “This discrepancy lies in the commence of dormancy, capacity to replicate and permeability to the surrounding microenvironment.” I believe that this manuscript would benefit from editing for grammar, word choice and sentence structure.

We have changed the sentence. Also, we have carefully rechecked for grammatical errors and revised the manuscript for better readability. The first author has grown up as native speaker in English speaking Hong Kong, nevertheless we do apologize as some errors may have skipped our attention.

2) I’m not fond of the use of “endorses” in the title.

We thank the comment from Reviewer #1, we have replaced the word with “enters”.

3) How does SpoT regulate dormancy? Does it repress SPI2 expression?

SpoT regulates ppGpp and (p)ppGpp levels that regulates ATP-dependent Lon protease activity targeting antitoxins of Type II toxin-antitoxin. The degradation of antitoxins releases the toxins that target and halt various cellular processes, ultimately inducing dormancy. SpoT regulates SPI-2 expression as reported by Fitzsimmons et al. 2020, therefore, we confirmed the Vac^-^Cyt^-^ induced by Δ*relAspoT* mutant has a comparable metabolic profile to that of wild type as indicated in the manuscript (Figure 3D, Right).

4) The figure legends should include all details of statistical analyses and numbers of cells and repetitions for each panel.

We have expanded the figure legends to include details on number of cells and analyses.

Reviewer #2: The study has as major strength the combination of previously used fluorescent reporters in new tool that allows to analyze simultaneosuly location and growth status of intracellular Salmonella. Apart from this technical advance, the study is conceptually weak, lacking much comparative discussion with prior work on Salmonella persisters in other cell models. Presentation of the data has also flaws, with some experiments described superficially and obvious controls missing. For example, the “colony forming unit plating” assay described in pag. 19 relates to the data shown in Fig. 4A, but in none of the two sites the cell line in which it was done, is specified. This assay involves a long-term infection (168 h, 7 days). It is not easy to imagine the experimental set-up when using epithelial cells as HeLa that are permissive for bacterial proliferation. Experimental details must be therefore precisely exposed. In general, there is clear overstatement in the conclusions due to the lack of more convincing data.

We appreciate the time and effort by Reviewer #2 in evaluating our manuscript in great detail. We are also thankful for the criticism, the insightful comments and the generous list of references provided, which are very helpful to improve the manuscript. We have significantly revised the manuscript taking into account the need to clarify the experimental setup used for the presented data. We have also included more literature discussion and most importantly, we performed substantial experiments to add more experimental data as requested. We hope that the revised version of the manuscript addresses the concerns of Reviewer #2.

**Part II – Major Issues: Key Experiments Required for Acceptance**

Reviewer #2: This study combines fluorescent reporters previously used to monitor subcellular localization of Salmonella (vacuolar, cytosolic) with a reporter that provides measure of replication rate based on a “Timer” dsRed variant.

The study does not have a clear starting hypothesis, but the application of this new dual reporter, named SINA, led to the authors to claim the existence of a “third” population of intracellular Salmonella that is intra-vacuolar and would remain in a dormant state form early infection times..
The first part of the study describing the new tools is technically convincing. However, the subsequent sections are much less solid and introduce concepts not supported by the data shown. Moreover, the study is not rigorous with the literature and it lacks many important controls being clearly biased by the data shown in macrophages. This phagocytic cell type is the sole comparator model used, leaving aside and omitting important observations in other models that provided valuable data on Salmonella intracellular persistence.

The study therefore forgets important observations in the Salmonella field regarding the capacity of this pathogen to establish a dormant state inside eukaryotic cells as well as the role of SpoT in controlling the intracellular virulence program. These omissions refer to the work performed in the fibroblast model, including: i) strong SPI-2 activity in non-growing intracellular Salmonella (Nunez-Hernandez et al., Infect Immun 2013, 2014) ; ii) production of several toxin-antitoxin modules by non-growing intracellular S. Typhimurium (Lobato-Marquez et al., Scientific Reports, 2015), none of which was tested here; iii) the selection of genetically table S. Typhimurium mutants with 3-4- logs higher capacity to persist inside the fibroblasts (Cano et al. Infect Immun 2003, 71: 3690-3698); and, iv) the role of SpoT, but not RelA, in producing ppGpp to stimulate activity of SPI-2 in intraphagosomal Salmonella (Fitzsimmons et al., mBio, 2020, 11:e03397-19).

We would like to thank Reviewer #2 for his/her time and we would like to express our gratitude for the appreciation on the novelty of the experimental tools.

We apologize having been limited in our description and discussion of our work focusing on epithelial cells and macrophages in our previous manuscript version. We decided to make this focus on a comparison with macrophages clearer in the revised version without neglecting the important published work on fibroblasts. Therefore, we greatly appreciate the list of references recommended by Reviewer #2. We have carefully consulted the suggested literature and incorporated the relevant references that enrich our discussion on *S*. Typhimurium dormancy. Along the same lines (and following a point raised by this reviewer), we have added experimental data using fibroblasts relating to these comments as described below.

From the work on the fibroblast model, we noted that *S*. Typhimurium was described as “nongrowing dormant bacteria” in the work of Nunez-Hernandez et al., Infect Immun, 2014 that referenced Cano et al., Infect Immun, 2001. Cano et al. reported on *S*. Typhimurium mutants that exhibit different intracellular proliferation capacities determined by comparing the cfu at 2 h and 24 h pi. Among the abovementioned dormancy fibroblast studies, the bacterial metabolic activity was not been tested by an inducible cassette (Helaine et al., 2010), a photoconvertible fluorescent protein (Liss et al., 2019) or fluorescent timer (Claudi et al., 2014). Therefore, the dormancy state described in the fibroblasts was based on the slow-growing nature of bacteria derived from the cfu ratio at 2 h and 24 h pi. We would like to mention that another fibroblast work by López-Montero et al., Autophagy, 2016 has reported that *S*. Typhimurium also extensively replicates within fibroblast in the first 24 h of infection (Fig 3A and Videos S1 to S3). This bacterial population is subsequently targeted by aggrephagy for degradation, and the fraction of bacteria not being targeted remains viable in the cell at 24 h pi. The intracellular proliferation of *S*. Typhimurium in fibroblasts in the first 24 h of infection is also in line with another work from Martínez-Moya et al., Research in Microbiology, 1998 (Fig 1B).

In sight of the substantial differences in metabolic activity and replication rates between the “nongrowing dormant” *S*. Typhimurium in fibroblasts and the “dormant” *S*. Typhimurium we report in our study, we intend to be vigilant and rigorous in making fair comparisons, while also adhering to the recent consensus statement by the antibiotic persistence research community (Balaban et al. Nature Reviews Microbiology, 2019). We have addressed these issues by carefully introducing the proposed literature of the reviewer and contextualizing it.

The study also lacks some rational justifications on why to choose exclusively the RelA-SpoT-DskA-Lon axis despite being forced to use “artificial conditions” in which SPI-1 is boosted by in-trans expression of HilA. Invading bacteria with high SPI-1 levels might be transiently prevented to correctly induced the SPI-2 intracellular phase.

We understand the concerns raised by Reviewer #2, and we would like to clarify that we have selected RelA-SpoT-DksA-Lon axis for its demonstrated functional role in *S*. Typhimurium dormancy in macrophages (see above). Under conditions of ectopic *hilA* expression, we observed a comparable amount of Vac^+^ population as compared to that without ectopic expression (compare Fig 1E and Fig 3D), indicating the ectopic *hilA* expression does not impede SPI-2 expression.

These are key experiments that should be addressed:

1) Ciprofloxacin survival assay (p. 19): The experiment involves incubation of infected cells in medium containing the drug. This design does not take into account that the bactericidal action of the drug can modify the physiology of intravacuolar bacteria to render it Vac- from a Vac+ status at the beginning of the treatment. Moreover, the Vac+Cyt+ epithelial cells contain both cytosolic bacteria undergoing rapid growth and intra-vacuolar bacteria with reduced growth rate. This Vac+Cyt+ cell population is included in the Material and Methods’ section (pag. 19) but not shown in Fig. 2D. So, it is uncertain whether or not was tested. Bactericidal effect might be different depending on the growth status inside the host cell. Furthermore, accessibility of the drug to the vacuolar compartments or the cytosol might not be similar. A more convincing experiment is to isolate bacteria from the different populations: Vac-Cyt- and Vac+Cyt- and further exposed to the drug in the test tube for 3 h and then do plating to count CFU next day.

We appreciate the suggestion made by Reviewer #2. In this regard, we would like to clarify the rationale behind our experimental design. To acknowledge and recapitulate the potential bottlenecks of antibiotic accessibility as well as evaluating the bactericidal effect under a more physiological condition, we decided to perform ciprofloxacin treatment during infection. The experimental approach using isolated bacteria is workable yet it is different from the performed experiment for two important features, the effective drug dosage that reaches the bacteria and the maintenance of bacterial physiology. As antibiotic molecules have individual physical and biochemical properties that could lead to different concentrations at specific sites, for example concentration at lipid droplets for some hydrophobic molecules. Direct combination of ciprofloxacin with isolated bacteria would not be able to address the efficacy of killing intracellular *S*. Typhimurium. Another fundamental aspect is the bacterial physiology, where *S*. Typhimurium isolated from host cells experience a drastic environmental shift from nutrient scarce vacuolar space to a culture medium, where nutrients are abundant. It is very likely that the *in vitro* condition promote dormancy exit and the return to a susceptible state, ultimately defying the objective to determine the antibiotic susceptibility of specific intracellular *S*. Typhimurium population. To explain our experimental strategy in a better way, we have decided to modify the respective text to clarify the populations enriched for cfu plating in our experimental design. In summary, we feel that the data from *in vitro* ciprofloxacin treatments would be difficult to interpret and would not be comparable to the *in cellulo* data, we decided against doing the proposed experimental work, also taking into account our needs to economize experimental time under the ongoing sanitary situation.

2) Considering the absolute of biochemistry in this study, it is recommended to tag in the chromosome a SPI2-specific gene (e.g. ssrB) and evaluate relative levels in the distinct populations (Vac-Cyt-, Vac+Cyt-, Vac+Cyt+). This must be done after sorting. This approach was useful to demonstrate SPI2 activity in non-growing Salmonella inside fibroblasts (Nunez-Hernandez et al., Infect Immun 2014).

We thank Reviewer #2 for this suggestion, and we have measured the SPI-2 activity by quantifying the abundance of PipB2 in distinct *S*. Typhimurium populations enriched by cell sorting by western blot. However, the amount of material we could collect in this experiment has remained too little to be detected by western blot with the available antibodies (data to be shared upon request) despite 6 hours of sorting (the maximum duration we could access the sorting under the current sanitary limitations), and sample degradation is of concern for further extended sorting duration.

To overcome these issues and to address the SPI-2 activity in different population, we decided to perform another experimental direction staining HeLa cells infected with JL177 and JL182 (the strain containing the HA-tagged PipB2) by indirect immunofluorescence using our anti-HA antibody to determine the abundance of HA-tagged PipB2 (FITC intensity) in infected cells harboring distinct *S*. Typhimurium populations, the gating strategy is identical to that described in Fig S3A .

Violin plot of abundance of PipB2-HA in dormant and SPI-2-expressing population in infected HeLa cells at 6 h pi.

We have repeated this experiment 2 times with identical results. As we could not do more replicates during the period of this revision, we share this data with the reviewers. We hope that this will be acceptable, as it has been a true challenge to execute the experimental additions under the current circumstances.

3) It is not obvious why the RelA-SpoT-DskA-Lon axis must be the only that operates to trigger generation of dormant Salmonella in epithelial cells. The work of Lobato-Marquez et al. (Sci Reports, 2015) showed that various TA systems operate in intracellular S. Typhimurium in both epithelial cells and fibroblasts. Unlike the relA, relAspoT and dskA mutants, none of the mutants lacking these TA systems were found to be impaired for invasion. So, no need to artificially manipulate SPI-1. These should be tested.

We appreciate the comments given by Reviewer #2. We wish to clarify that we do not make any direct or indirect claims in support of the notion that “the RelA-SpoT-DskA-Lon axis must be the only one that operates to trigger generation of dormant Salmonella in epithelial cells”. We have carefully studied Supplementary Fig. S4 of the work of Lobato-Marquez et al. (Sci Reports, 2015), which did not include Δ*relA*, Δ*relAspoT* and Δ*dksA* mutants. We have chosen the RelA-SpoT-DksA-Lon axis for its demonstrated functional role in *S*. Typhimurium dormancy in macrophages (see our comment about this choice above). Of note, the work of Pizarro-Cerdá and Tedin (Molecular Microbiology, 2004) has demonstrated that Δ*relA*Δ*spoT* mutant displays a reduced invasiveness phenotype that is reduced by *hilA* ectopic expression (as shown in Figure 6 of the work by Pizarro-Cerdá and Tedin). We decided not to test TA systems in this version of the manuscript, as it is at the moment out of the scope of this work. Such an exploratory research line is nevertheless worthy of investigating in the future.

**Part III – Minor Issues: Editorial and Data Presentation Modifications**

Reviewer #2: - Abstract (lines 14-16): In no part of the study the authors show that the dormant bacteria Vac-Cyt- express SPI-2 al later infection times. The next sentence assumes that dormant bacteria could have “lost” SPI2 activity, which is also not demonstrated by any experiment.

We thank the comment made by Reviewer #2. We would like to clarify that we have demonstrated the SPI-2 expression by dormant *S*. Typhimurium at 24 h pi in Fig 2G (now Fig 2H). We have rephrased the following point on SPI-2 activity by precising the SPI-2 effector secretion but not the SPI-2 expression that is demonstrated in Fig 3A.

- Pag.3 Importance of the work: Line 4 (We found….). This reviewer does not agree with this statement. It assumes, but it does not prove, that formation of dormant bacteria in epithelial is the one that operates in macrophages. Moreover, TacT is vaguely cited, not functionally tested and no phenotypic test is done with any other TA systems reported to be induced by dormant S. Typhimurium.

We thank the concern raised by Reviewer #2, we have rephrased this statement and specified the pathway as stringent response pathway to avoid misunderstanding. We have cited and described the work on TacT in both the introduction and discussion in the context of TA systems. We did not emphasize TacT or alternative TA systems because we found Lon, a Type II TA system regulator, is dispensable for *S*. Typhimurium dormancy in epithelial cells (TA system review by Wen et al. Pathogens and Disease, 2014).

- Fig. 1C. The colours expected with the two reporters are shown in two cells but the respective names of the reporters are missing.

We would like to thank Reviewer #2 for catching this issue. We have added descriptions of the localization and replication rate modules on Fig 1C.

- Figure 4A. Discussing these data the authors do not cite the work published in 2003 showing cultivability of dormant S. Typhimrium recovered from fibroblasts after one-week infection (Cano et al. Infect Immun.)

We have studied the reference recommended by Reviewer #2, we have made revisions in line 350-351 that cites the reference on cultivable bacteria identified after 6 days of infection.

- Although the authors insist that regulation of persisters’ formation might differ among distinct cell types, no comparative analysis is shown. This is extremely relevant considering that the studies in fibroblasts as well as those of Helaine’s group in macrophages have linked SPI2 activity to dormant intracellular Salmonella. The only exception is Fig. S8 with THP1 macrophages, in which up to 70% of the Vac-Cyt- population is shown. Where are then the dormant bacteria reported by Helaine’s group present in these macrophages?|

We are aware of the different “persisters” defined and reported in different model systems by different teams. Also, we have taken the comments above into account with regards to fibroblasts. Therefore, we have substantially revised our discussion on the differences between the “persisters” from different cell types in Discussion paragraph 3 (Lines 340-360).

Furthermore, we have performed experimental analysis on fibroblasts and macrophages at 1 h, 6 h and 24 h using our tools (Fig S8 and S9). We wish to point out that non-dividing dormant *S*. Typhimurium reported by Helaine’s team was detected between 18 h to 24 h pi, which is not detected at 6 h pi. At 24 h pi, we observed a population of infected cells being Vac^-^Cyt^-^, which has a distinct Timer profile as compared to that of Vac^+^Cyt^-^ (Fig S9), hinting at the presence of a bacterial population similar to that described by the Helaine’s team.

- Supplementary figure S4 clearly shows that the Vac-Cyt- population in HeLa decreases over time, chaining from 37.7% at 2 hpi to 7.41% at 6 hpi. The Vac+Cyt- population however remains rather stable, moving from 60% to 76% at 6 hpi. Authors should comment on the significance of this decrease.

We would like to clarify that the gating was defined based on the Vac and Cyt signal intensity at 1 h pi (shown in Fig 1E and S2). The “decrease” described by Reviewer #2 refers to the increase in signal output from basal level (ie 1 h pi) up to 6 h pi as P*_ssaG_* promoter is active only after bacterial entry. The percentage difference at different timepoints is due to the signal output from SINA when *S*. Typhimurium adopts various lifestyles. The Vac^+^ populations (Vac^+^Cyt^-^ and Vac^+^Cyt^+^) increased for a total of 30.36%, which matches closely with the 30.29% reduction in Vac^-^Cyt^-^ described by Reviewer #2.

- Description or discussion of some data do not reflect accurately the results shown with not alternatives being considered. An example is the “loss” of SPI2 expression in the Vac-Cyt- population, mentioned repeated times. The authors do not discuss about the possibility of these bacteria not triggering SPI2 expression at any time of the infection and remaining frozen at some early stage of SCV biogenesis.

We thank the concerns raised by Reviewer #2. In the revised manuscript, we have included immunofluorescence staining against markers of different stages of SCV biogenesis (RAB5, RAB7 and RAB11), which are shown to be not localized to the dormant SCV, suggesting the Vac^-^ resides in an unknown compartment. This has been also further substantiated by our CLEM studies. We have discussed this phenomenon in the discussion of the submitted manuscript (line 327-332). Regarding the SPI-2 expression in Vac^-^Cyt^-^ population at later time points, we did observe the SPI-2 expression in this population as demonstrated in Fig 2H.

- Another missing discussion is that related to the possibility of Vac-Cyt- intracellular bacteria being masked by other populations the same infected cell. Heterogeneity regarding epithelial cells harbouring Vac+ and Cyt+ bacteria was first characterized by Knodler’s lab and it is also evident in this study. So, Vac-Cyt- bacteria can be also present in some of the infected populations assigned as Vac+Cyt- and Vac+Cyt+.

We thank the suggestion offered by Reviewer #2. We have added a discussion on the Vac^-^Cyt^-^ population not co-existing with bacteria of other lifestyles (line 402-404), which has been observed via fluorescence microscopy (Fig 2F). From our experience, even though it is possible that the Vac-Cyt- population co-exists with others, it seems that this phenomenon is rare enough, and we haven’t detected this by fluorescence microscopy in our setup.

- The data shown compare the Vac-Cyt- population in other cell lines such as Caco-2 and THP1 are surprising in the sense that they show a much larger Vac-Cyt- populations (34% and 70% at 6 hpi, respectively for each cell line). This reviewer wonders why these cell lines were not selected better than HeLa for the purpose of demonstrating the existence of such dormant bacteria. An additional cell type missing are fibroblasts, in which dormant population predominates versus proliferating bacteria. The SINA tool could be of impressible utility in these alternative models.

We acknowledge the comment made by Reviewer #2. Our design of SINA is tailored to distinguish the *S*. Typhimurium intracellular lifestyle in epithelial cells, where we constructed two modules to depict the bacterial localization and replication, the two remarkable features of *S*. Typhimurium lifestyles in enterocytes. We did not select polarized Caco-2 as the major model due to its limited scalability from trans-well usage that is technically restraining the study of the scarce dormant population. Another reason is that polarized Caco-2 cells require strong sheering force for cell detachment, which is sub-optimal for cell enrichment and re-plating. On the other hand, we took the suggestion from Reviewer #2 and have supplemented the data on fibroblast using 3T3 as the model (Fig S8).

- Genetic background (genotype) of the S. Typhimurium wild-type strain in which plasmid were inserted should be indicated in the Table of strains, no just in the text.

We thank the reminder by Reviewer #2 and we have revised Table S1.

- Lines 3 and 5, 3rd paragraph, page 4. Include references.

We thank the careful reading of Reviewer #2 and we have revised the text accordingly.

- Line 12, 3rd paragraph, page 4. Provide more details about the specific metabolic activity.

We thank the careful reading of Reviewer #2 and we have revised the text accordingly.


- Line 2, 1st paragraph, page. Reference of Lobato-Marquez et al. (Sci Rep, 2015) is missing when referring to TA systems.

We thank the careful reading of Reviewer #2, we would like to highlight that the sentence is referring to TA systems demonstrated to modulate *S*. Typhimurium lifestyle in macrophages.

- The manuscript was not line numbered. To be corrected in future versions.

As suggested, we have added the line numbering to make the revision easier.

- The distinct panels of the videos (S1-to-S3) are not labelled for the respective channel shown. Moreover, the lower panel is pixelated in grey scale with no clear image(s) seen.

We have included the labelling for each of the channels.

- Reference 13 is incomplete. It has no journal information.

We thank Reviewer #2’s careful checking, we have updated the reference.

- 2nd paragraph, page 5: TacT is not introduced first time it is cited.

We thank the careful reading of Reviewer #2, we have included the citation (line 52).

- Pages 7-9. Figure S3 is cited after S4 and S5.

We have cited Fig S3 in line 92, before citing S4 in line 120.

- Fig 2C: use better contrasting colours for the bars shown in middle panel.

We thank the suggestion of Reviewer #2, we intend to adopt an identical color code for smURFP as Fig 2B to avoid confusion.

- Fig 2D: What are it the lines connecting the percentage of Dormant and Vac+Vac- populations? Are they independent biological replicas? The legend refers to n = 3.

We thank the careful reading of Reviewer #2, we have revised the text accordingly in the new Figure 2I.

- Figure 3B: it lacks staining of representative bacteria from Vac+Cyt- and Vac-Cyt+ populations. Labelling of early-medium markers (Rab5, Rab7) is also recommended to dissect the exact nature of compartment in which these dormant are.

We thank the suggestion of Reviewer #2, we have supplemented the immunofluorescence staining of RAB5, RAB7 and RAB11.

- What is Fig. 4B? Please provide a scheme of the experimental design. The way it is described in the legend is not clear at all.

We thank the concern raised by Reviewer #2, Figure 4B is our model for the proposed implications of enterocyte-borne dormant *S*. Typhimurium that we cover in the discussion. It is not related to an experiment.

- Line 1, 4th paragraph, page 11. First sentence seems incorrect. Please, rephrase.

We thank the careful reading of Reviewer #2, we have revised the text (line 265) accordingly.

- Page 13. Discussion refers to “first” S. Typhimurium persisters reporter in macrophages. Chronologically, the last paragraph is not correct. See comments above about earlier studies in fibroblasts.

We thank the reminder by Reviewer #2, we have revised the text to specify “antibiotics persisters”.

- Discussion is unnecessary long. It should be shortened by at least one page.

We thank the suggestion by Reviewer #2, we have carefully revisited the Discussion section trying to be as focused as we can. Also, we are now covering the literature suggested by this reviewer. To shorten a bit, we also deleted the last paragraph of the discussion, as it provides a rather broader perspective.

Reviewer #3: The authors use Salmonella infection as a model to address important questions about bacterial growth in different compartments of host cells. They also ask where Salmonella persisters colonize host cells, and how this is regulated. They develop a new probe (SINA) that indicates both bacterial localization and also metabolic activity at the same time. They propose that a population of dormant bacteria occupies an uncharacterized compartment and can be re-activated upon favorable growth conditions. The strength of this study is the development of new methods and they test these both in vitro and in vivo. This is an interesting study and the results are promising, but I have some questions about the probe and the interpretation of the data.

We are very grateful for Reviewer #3’s time in evaluating our manuscript and we are thankful by the Reviewer’s positive feedback on the novelty of the experimental approaches and the importance of our work.

**Part II – Major Issues: Key Experiments Required for Acceptance**

Reviewer #3: -The new SINA probe is being used to identify a population of dormant bacteria. Are there any other bona fide markers/probes that can be used in tandem to confirm what the authors are concluding? Perhaps RT-PCR or mass spec analysis of cell sorted bacteria would help?

We thank the experiments suggested by Reviewer #3, we have tried to perform western blot targeting SPI-2 effectors PipB2 with sorted bacteria population, however this is limited by the amount of material we could retrieve from cell sorting. We have addressed this by an alternative approach by staining against HA-tagged PipB2 in infected HeLa cells and analyzed by flow cytometry. We determined the abundance of HA-tagged PipB2 (FITC intensity) in infected cells harboring distinct *S*. Typhimurium populations using a gating strategy identical to that described in Fig S3A .

Figure: Violin plot of abundance of PipB2-HA in dormant and SPI-2-expressing population in infected HeLa cells at 6 h pi.

We have repeated this experiment 2 times with identical results. As we could not do more replicates during the period of this revision, we share this data with the reviewers. We hope that this will be acceptable, as it has been a true challenge to execute the experimental additions under the current circumstances.

Also, we have performed electron microscopy on the dormant bacteria within a vacuolar compartment. In addition, we have characterized the dormant population as described in figure 2.

-The new probes are plasmids. Is it stably integrated into the bacterial chromosome? If not, what evidence is there that plasmid loss is not occurring? Again, maybe RT-PCR would help.

The SINA system is plasmid-borne and not being integrated into the bacterial chromosome. The ability to grow on ampicillin agar plates, even after sorting and replating cells that are further cultured for several days, serves as an indicator of the reporter plasmid remains present in the bacteria, where AmpR is the selection marker on the reporter plasmid. If the plasmid was lost, we would not be able to obtain the data for most of the results shown in figure 2.

-The authors refer to bacteria lacking expression of ssaG (SPI-2 T3SS component, used as marker of vacuolar) and uhpt (hexose 6-phosphate: phosphate antiporter; used as marker of cytosolic bacteria) and having a low metabolic activity as being “dormant”. An alternative interpretation is that these bacteria merely lost the plasmid containing the SINA probe. Or do the bacteria go to a non-acidified compartment without hexose 6-phosphate?

We appreciate the query raised by Reviewer #3. The dormant bacteria enriched by cell sorting can regrow on ampicillin agar plates containing ampicillin, indicating the reporter plasmid is not lost during the 6h infection time course. It is possible that the dormant bacteria reside in a non-acidified compartment without hexose-6-phosphate, which aligns with our observation on the lack of late endosome markers on the dormant SCV (Fig 2D and 2E). This is also further substantiated by our CLEM data.

-Supplemental Movie 1. What is the movie supposed to be showing? It might help to have a separate figure with frames from the movie where bacterial signals and populations are highlighted.

The supplementary movie is intended to show the signal output from SINA in vacuolar bacteria. We have accordingly incorporated a panel of selected frames to depict the highlighted events (Fig S4B and S4C).

-Figure 1D, the labeling is confusing, and not consistent with figure legend. For example, “6h SPI-2” not the same as “vacuolar”. When I first read this I thought a mutant was used, so confused. The addition of colour only to the merged channel makes the situation worse. Importantly, what is the conclusion from this figure? Are vacuolar bacteria less metabolically active than those in the cytosol? Is there a population of fast and slow growing bacteria in the cytosol?

We appreciate the suggestion made by Reviewer #3, and we have modified accordingly the color code and legends of the figure panels. The main idea of the figure is to demonstrate the functionality of the SINA system in distinguishing vacuolar and cytosolic *S*. Typhimurium using both microscopy and flow cytometry.

-Figure 1E. Why are there only Vac+Cyt+ bacteria, and not Vac-Cyt+ bacteria? What does this suggest about the probe, or potentially the bacterial entry to the cytosol? The authors should discuss this point.

We thank the query raised by Reviewer #3 regarding the bacteria population. We have observed Vac^+^Cyt^-^ and Vac^-^Cyt^+^ *S*. Typhimurium via microscopy as depicted in Figure 1D. In Figure 1E, infected cells were analyzed by flow cytometry, the Vac^+^Cyt^+^ population refers to infected cells harboring both vacuolar and cytosolic bacteria (Fig 1D, Bottom). We hope that it comes out in the way we wrote the text.

-pg8 “This revealed that the Vac-Cyt- S.Typhimurium exhibit a similar replication rate (S5A Fig) but a reduced metabolic activity (S5B Fig) compared to Vac+Cyt- S. Typhimurium as depicted by the green:red ratio and slope of Timerbac plot, respectively”. This is a confusing conclusion: how can the authors propose a similar replication rate with reduced metabolic activity? It seems likely the probe is providing an artefactual lack of signal, or the bacteria are in an unusual location such as the cell surface.

We thank the question raised by Reviewer #3 on the statement made. The statement was made from the Timer^bac^ readout, vacuolar *S*. Typhimurium undergoes one to two rounds of replication until 6 h pi, which is a heterogenous phenomenon that is not picked up by Timer^bac^, unlike the hyper-replication of cytosolic bacteria. The bacteria cannot be at the cell surface as they are killed there and washed away in our experimental setup (see also next comment).

-are antibiotics present in the medium during experiments? The authors point out that antibiotics affect dormancy…. Is this driving observed phenotypes? What happens when gentamicin is removed from the medium?

We appreciate the concerns raised by Reviewer #3 on the impact of antibiotics. We have observed the conventional high-low Gentamicin treatment for killing extracellular bacteria would lead to an elevated level of Vac^-^Cyt^-^ population, to avoid such artefacts, we only supplement Gentamicin to the medium at 3 h pi, which is a time point after the formation of Vac^-^Cyt^-^ population as shown in Fig S6A.

-Movie S3. There does not appear to be any signals. What are we supposed to be looking at?

Movie S3 depicts the dormant *S*. Typhimurium that is Vac^-^Cyt^--^, only the Timer^bac^ channels give visible signals in this population.

-pg9, “With SINA1.5, we observed that approximately half of the Vac-
S. Typhimurium did not respond to arabinose induction at designated time intervals
(Fig 2C). Combining this with our observations on the reduced metabolism (S5B Fig),
we were able to put forward the first evidence to propose that the Vac-Cyt- S.
Typhimurium adopts a dormant state (coined as dormant S. Typhimurium hereafter)”. Is arabinose cell permeable? Could a lack of induction simply reflect a disconnection of bacteria in unusual vacuoles from the endocytic pathway?

We appreciate the concerns raised by Reviewer #3. This arabinose induction assay has been used before to study dormant bacteria as we refer to in the manuscript. Nevertheless, arabinose is not permeable to the mammalian cell membrane. The lack of induction could be attributed to the disconnection of endocytic pathway or the bacteria undergoing dormancy. To differentiate the two scenarios, we also rely on the readout from Timer^bac^ to determine the Vac^-^ population is harboring a reduced metabolic rate (Fig S5B). We have clarified the writing on this.

-pg10 “Therefore, we conclude that dormant S.Typhimurium are localized within a unique membrane-bound compartment distinct from the conventional SCV”. How can the authors conclude the bacteria are present in a cellular compartment if no markers are present? The authors should consider using correlative electron microscopy to visualize what they are calling a dormant population within cells. Staining for ubiquitinated proteins (typically seen on cytosolic bacteria) should also be considered.

We appreciate the suggestion made by Reviewer #3. We have performed correlative light electron microscopy to confirm that the dormant *S*. Typhimurium we described is intracellular and encapsulated by a host membrane. We also did these experiments using our FIBSEM pipeline published before, but during the time of revision, there were some technical issues that resulted in unfocused images. Therefore, we show the serial sectioning TEM in our paper (that has actually higher resolution).

-Related to above, what is the morphology of the dormant bacteria? Upon entry to the cytosol some Salmonella increase in size and decrease in antibody staining… does this type of visual analysis suggest dormant bacteria are in the cytosol?

We appreciate the suggestion made by Reviewer #3. We have not quantified the morphological details of the dormant bacteria. To confirm the subcellular localization of dormant *S*. Typhimurium, we have performed correlative light electron microscopy (Fig 2B) and digitonin assays (Fig S3A-F) to demonstrate its vesicular localization. This clearly highlights the vacuolar localization of our dormant population.

-Are dormant Salmonella found in cells that have Salmonella-induced filaments (Sifs)? Or those that don’t? This would be interesting to know since bacteria that make Sifs are associated with growth of the bacteria.

We thank the question raised by Reviewer #3, we did not observe dormant *S*. Typhimurium to co-exist with SPI-2 expressing bacteria. As SPI-2 effectors are required for the formation of SIFs, it is unlikely to have dormant bacteria in cells harboring SIFs. Moreover, our staining for LAMP1 does not show the presence of SIFs or similar structures in cells containing dormant bacteria at 6 h pi.

-Figure 4. Dormant bacteria were isolated by flow sorting… can the authors perform microscopy to show what was purified? Importantly, did the collected bacteria bear the selectable marker of the SINA probe?

We understand the concerns of Reviewer#3 in this regard, although we don’t have microscopy images of sorted bacteria, we have performed flow cytometry to determine the purity of the enriched infected cells harboring Vac^-^Cyt^-^ population (FACS plot displayed below). The isolated population bears the selection marker as they remain viable and cultivable on Amp agar plate.

**Part III – Minor Issues: Editorial and Data Presentation Modifications**

Reviewer #3: -Title: I’m not sure “endorses” is the right word to use…

We thank the comment from Reviewer #3, we have replaced the word with “enters”.

-Short title: perhaps “in enterocytes” is better?

We thank the suggestion by Reviewer#3, we have made the changes accordingly.

-Supplemental movies: it would help to label the frames so we know what we are looking at.

We thank the suggestion by Reviewer#3, we have addressed it accordingly in Fig S4B and S4C.
